# Impact of high‐grain diet feeding on mucosa‐associated bacterial community and gene expression of tight junction proteins in the small intestine of goats

**DOI:** 10.1002/mbo3.745

**Published:** 2018-10-24

**Authors:** Junhua Liu, Chunxu Xue, Daming Sun, Weiyun Zhu, Shengyong Mao

**Affiliations:** ^1^ Jiangsu Key Laboratory of Gastrointestinal Nutrition and Animal Health, Laboratory of Gastrointestinal Microbiology, College of Animal Science and Technology Nanjing Agricultural University Nanjing Jiangsu Province China

**Keywords:** goat, high‐grain diet, mucosal bacteria, small intestine, tight junction protein

## Abstract

The objective of this study was to investigate the impact of a high‐grain (HG) diet on the microbial fermentation, the composition of the mucosa‐associated bacterial microbiota, and the gene expression of tight junction proteins in the small intestine of goats. In the present study, we randomly assigned 10 male goats to either a hay diet (*n* = 5) or a HG diet (56.5% grain; *n* = 5) and then examined changes in the bacterial community using Illumina MiSeq sequencing and the expression of tight junction proteins using qRT‐PCR in the mucosa of the small intestine. The results showed that HG diet decreased the luminal pH (*p* = 0.005) and increased the lipopolysaccharide content (*p* < 0.001) in the digesta of the ileum, and it increased the concentration of total volatile fatty acids in the digesta of the jejunum (*p* = 0.015) and ileum (*p* = 0.007) compared with the hay diet. MiSeq sequencing results indicated that the HG diet increased (FDR = 0.007–0.028) the percentage of the genera *Stenotrophomonas*,* Moraxella*,* Lactobacillus*, and *Prevotella* in jejunal mucosa but decreased (FDR = 0.016) the abundance of *Christensenellaceae R7* group in the ileal mucosa compared with the hay diet. Furthermore, the HG diet caused downregulation of the mRNA expression of claudin‐4, occludin, and ZO‐1 in jejunal and ileal mucosa (*p* < 0.05). Collectively, our data suggested that the HG diet induced changes in the relative abundance of some mucosa‐associated bacteria, in addition to downregulation of the mRNA expression of tight junction proteins in the small intestine. These findings provide new insights into the adaptation response of the small intestine to HG feeding in ruminants.

## 1 INTRODUCTION

To increase the growth rates or milk yields, goats or dairy cattle are often fed high‐grain (HG) diet in commercial ruminant production system (Plaizier, Krause, Gozho, & McBride, 2008). Changes in feeding strategies from forage to grains can result in an unhealthy accumulation of organic acids in the rumen (Kleen, Hooijer, Rehage, & Noordhuizen, 2003). Ruminants fed a HG diet may reduce the time spent chewing, thereby decreasing salivary secretion and the neutralization of volatile fatty acids (VFA), leading to a lower ruminal pH, which can depress feed intake and fiber digestion, negatively affect the health of ruminants, as well as alter milk composition and meat quality (Plaizier et al., 2008).

In recent years, nutritionists have paid great attention to HG feeding and gastrointestinal health in ruminants. However, most of the studies mainly focused on how HG feeding affects bacterial community and health of rumen and hindgut in dairy cows and goats (Liu, Xu, Zhu, & Mao, 2014; Plaizier, Khafipour, Li, Gozho, & Krause, 2012; Ye, Liu, Feng, Zhu, & Mao, 2016). Little information is available about the bacterial community and health of the small intestine in ruminants during HG feeding. The small intestine not only is the functional organ responsible for nutrient digestion and absorption, but also is an important barrier and immune organ in the ruminants. Its health status has an important role in maintaining animal performance (Branco, Harmon, Bohnert, Larson, & Bauer, 1999). When ruminants are fed a HG diet, excessive undigested starch or VFA flows from the rumen into the small intestine (Moharrery, Larsen, & Weisbjerg, 2014), which may induce imbalance of bacterial communities (Russell & Rychlik, 2001) in the small intestine. This imbalance can finally cause enterotoxaemia, leading to sudden death of animal (Glock & DeGroot, 1998; Russell & Rychlik, 2001). Therefore, the health of ruminants depends on developing nutritional strategies in order to maintain a well‐balanced microflora in the small intestine. Our previous study has showed that HG feeding altered the composition of digesta‐associated bacteria in the ileum of goats (Mao, Huo, & Zhu, 2013). However, little information is available on changes in the composition and diversity of the mucosa‐associated microbiota and mucosal health in the small intestine of ruminants fed a HG diet. Mucosa‐associated bacteria in the small intestine play a critical role in regulating the barrier function and immune system (Kelly et al., 2016). Thus, maintenance of the well balance of the mucosa‐associated microbiota of small intestine in ruminants is extremely important for health and performance of goats.

Here, we hypothesized that feeding goats a HG diet could affect the microbial fermentation, composition and diversity of mucosa‐associated bacterial communities, and mucosal health of jejunum and ileum. Therefore, the objective of this study was to investigate the changes in the composition and diversity of jejunal and ileal mucosa‐attached microbiota in goats fed HG diets. In addition, we evaluated the impact of HG diets on the mRNA expression of mucosal tight junction proteins of the jejunum and ileum in goats. These findings will be important for better understanding of composition of mucosa‐associated microbiota in the small intestine and how HG feeding affects the mucosal health of entire gastrointestine of the ruminants.

## 2 MATERIALS AND METHODS

### 2.1 Ethics statement

The experimental protocol was approved by the Animal Care and Use Committee of Nanjing Agricultural University, Jiangsu Province, China.

### 2.2 Animals and experimental design

The detail of animal experimental design has been reported in the previous study (Liu et al., 2014; Ye et al., 2016). Ten, 8‐month‐old male goats (Boer × Yangtze River Delta White) were adapted to a whole hay diet for 14 days and then randomly divided into two different groups. One group was fed a hay diet (hay, 4% concentrate and 96% hay, *n* = 5), and the other group was fed a HG diet (75% concentrate and 25% hay, *n* = 5). The experimental period was 7 weeks. Daily rations (900 g/animal) were divided into two equal portions. One portion was offered at 8:00 hr, and the other was offered at 17:00 hr. Each goat was placed in a separate pen (1.2 × 1.2 m), with free access to water.

At the beginning of the feeding trial, there were no significant between‐group differences in the body weights of the goats (27 ± 3 kg). The diets were designed according to the nutrient requirements of goats (NY/Y816–2004; Ministry of Agriculture of China, 2004). The metabolic energy in the hay group was 8.32 MJ/kg dry matter, which permitted a maintenance requirement of 30 kg goat. The metabolic energy in the HG group was 11.56 MJ/kg dry matter, which permitted a growth rate of 200 g/day of goats. Table S1 presents the nutrient composition of the diets in the study.

### 2.3 Sample collection

On day 50, all the animals were slaughtered 4 hr after morning feeding. Jejunal and ileal tissue samples were then collected and washed three times using ice‐cold phosphate‐buffered saline (pH 7.4) within 5 min after slaughter. The jejunal and ileal mucosal samples were scratched using a glass slide and immediately placed in liquid nitrogen. The samples were then stored at −80°C until further processing for RNA extraction and microbial DNA extraction. Digesta of the jejunum and ileum was collected, and the pH was determined. All the samples of digesta were then homogenized thoroughly and divided into two parts. The first part of the homogenized digesta was mixed thoroughly with a double volume of distilled water and was centrifuged at 2,000 *g*. The supernatant was then collected and stored at −20°C for determination of the VFA, lactate concentration, and amylase activity. The other part of the homogenized digesta was mixed with an equal volume of endotoxin‐free water and was centrifuged at 13,000 *g* for 40 min. The supernatant was collected using a lipopolysaccharide (LPS)‐free filter. The filtrate was transferred to a sterile glass tube and heated at 100°C for 30 min. It was then placed in freezer at a −20°C for later analysis of LPS.

### 2.4 Measurements of parameters of jejunal and ileal fermentation

The pH of the jejunal and ileal digesta was measured immediately at the time of extraction using a pH meter (HI 9024C; HANNA Instruments, Woonsocket, RI, USA). The VFA and lactate concentrations were determined by methods described previously (Barker & Summerson, 1941; Ye et al., 2016). The LPS content was analyzed using a chromogenic end point Tachypleus amebocyte lysate assay kit (Chinese Horseshoe Crab Reagent Manufactory, Xiamen, China), as reported previously (Mao et al., 2013). The amylase activity was detected using an Amylase Activity Assay Kit (Jiancheng Bioengineering Institute, Nanjing, China).

### 2.5 Mucosal microbial DNA extraction

The DNA of all the mucosal samples was extracted using a QIAamp Fast DNA Stool Mini Kit (Qiagen, Hilden, Germany). The mucosal samples were placed in a centrifuge tube, and 1 ml of InhibitEX buffer was added to each sample and then homogenized by vortexing for 1 min. The resulting liquid was incubated for 5 min at 70°C. The lysis temperature was increased to 95°C, and the cells were then vortexed for 15 s. The liquid was centrifuged for 1 min to produce a pellet of particles. The supernatant was pipetted into a centrifuge tube with Proteinase K (15 μl) and AL buffer (200 μl) and then heated at 70°C for 10 min. The lysate was then placed in 200 μl of ethanol, followed by washing with 500 μl of AW1 and AW2 buffer. Subsequently, the solution was centrifuged to obtain the supernatant. The buffer (200 µl) was then added to the supernatant, which was left at room temperature for 1 min, followed by elution of DNA from the sample. The concentration of DNA was determined using a NanoDrop 2000 spectrophotometer (Thermo Fisher Scientific). The extracted DNA samples were stored at −80°C until further processing.

### 2.6 PCR amplification and DNA illumina MiSeq sequencing

The following primers were used for amplification of 16S rRNA from the V3‐V4 region: 338F (5′‐barcode‐ ACTCCTRCGGGAGGCAGCAG)‐3′ and 806R (5′‐GGACTACCVGGGTATCTAAT‐3′) (amplicon length: ~470 bp). The cycling parameters for the PCR were as follows: denaturation at 95°C for 2 min, 25 cycles at 95°C for 30 s, annealing at 55°C for 30 s, 72°C for 30 s, and extension at 72°C for 5 min. The PCR products were separated using 2% agarose gels, and an AxyPrep DNA Gel Extraction Kit (Axygen Biosciences, Union City, CA, USA) was used to purify. Amplicons were quantified using the QuantiFluor‐ST (Promega, Durham, NC, USA) device. An Illumina TruSeq DNA Sample Preparation Kit (Illumina, San Diego, CA, USA) was used to construct a sequencing library from the PCR products. Illumina TruSeq PE Cluster and Sequencing by Synthesis kits (Illumina) were used to perform cluster generation, template hybridization, isothermal amplification, linearization, blocking and denaturation, and hybridization of the sequencing primers. Paired‐end sequencing of 2 × 250 bp was performed to sequence all the libraries on the Illumina MiSeq platform (Caporaso et al., 2012).

### 2.7 Sequencing data analyses

The data were analyzed using the Quantitative Insights into Microbial Ecology (QIIME; version 1.8.0; https://qiime.org /) software package (Caporaso et al., 2010). The trimmed sequence reads were distributed to every sample on the basis of barcodes. High‐quality sequences of >250 bp without ambiguous “N” bases and an average base quality score >25 were identified and used in the subsequent analysis. Chimeric sequences were filtered using UCHIME (Edgar, 2010). Using UPARSE (https://drive5.com/uparse/), operational taxonomic units (OTUs) were clustered based on a threshold of 97% sequence similarity. The OTU sequences were classified at the taxonomic level using the RDP Classifier (Wang, Garrity, Tiedje, & Cole, 2007) and aligned using the Silva reference database (SILVA 128) (DeSantis et al., 2006) and PyNAST (Caporaso et al., 2010). The default index was set by QIIME. The abundance‐based coverage estimator (ACE), chao1 estimator, Shannon's diversity index, and Simpson's diversity index were used to calculate the richness and diversity of the bacterial community. The FastTree tool (Price, Dehal, & Arkin, 2009) was used to build a phylogenetic tree. A principal coordinate analysis (PCoA) (Lozupone & Knight, 2005) of Bray–Curtis distance matrix was performed to measure the strength of the sample cluster. Significant differences between the two groups were calculated based on a Bray–Curtis distance matrix‐based analysis of molecular variance (AMOVA) using the MOTHUR program (vs. 1.39.0) (Schloss et al., 2009).

### 2.8 Mucosal RNA extraction and real‐time PCR

The jejunal and ileal mucosal RNA was isolated using TRIzol (Takara Bio, Otsu, Japan), as described by Chomczynski and Sacchi (1987) and quantified using a NanoDrop spectrophotometer (Thermo Scientific Inc., Wilmington, DE, USA). Aliquots of RNA samples were subjected to electrophoresis on a 1.4% agarose–formaldehyde gel to verify integrity. A PrimeScript RT reagent kit (Takara Bio, Otsu, Japan) was used for reverse transcription of RNA. The primers for tight junction proteins (claudin‐1, claudin‐4, occludin, and zonula occludens‐1 [ZO‐1]) have been described previously (Liu, Xu, Liu, Zhu, & Mao, 2013) and are listed in Table S2. The primer for the housekeeping gene was designed to recognize and amplify a conserved nucleotide sequence encoding 18S rRNA. The cDNA sequence was distinguished using a basic local alignment search tool (National Center for Biotechnology Information, Bethesda, MD, USA). The primers were designed by the Primer 5.0 (Whitehead Institute, Cambridge, MA, USA). Tight junction proteins, 18S rRNA, were analyzed using the ABI 7300 real‐time PCR system (Applied Biosystems, Foster, City, CA, USA), with fluorescence detection of SYBR Green (Takara Bio, Otsu, Japan). The reaction conditions were 95°C for 30 s, 40 cycles at 95°C for 5 s, and then 60°C for 31 s. Then, 1–10 ng of cDNA was added to the 2× SYBR Green mix (Takara Bio), and primers (200 nmol/l of each primer), Rox Reference Dye (50×), and nuclease‐free water were added to the mixture to reach a volume of 20 μl. The target gene expression normalized to that of the housekeeping gene was calculated using the $2^{-\Delta \Delta CT}$ method.

### 2.9 Statistical analyses

The statistical calculations were analyzed using the SPSS software package (SPSS vs. 20, SPSS Inc., Chicago, IL, USA). Fermentation parameters, bacterial diversity and richness data, and mRNA expression of tight junction proteins were analyzed by an independent *t* test. The relative abundance of microbial phyla, genera, and OTUs was determined using the nonparametric Kruskal–Wallis test. All *p*‐values of the bacterial community obtained by the nonparametric Kruskal–Wallis test were corrected for the false discovery rate (FDR). A value of *p* < 0.05 was considered significant.

## 3 RESULTS

### 3.1 pH, concentrations of VFA, lactate, and LPS, and amylase activity in the jejunal and ileal digesta

As shown in Table 1, in jejunal digesta, the HG diet increased the concentrations of total VFA (TVFA) (*p* = 0.015), propionate (*p* = 0.008), butyrate (*p* = 0.004), and isobutyrate (*p* = 0.035) and decreased the levels of lactate (*p* = 0.008), as compared with the hay diet. In ileal digesta, the HG diet decreased the luminal pH as compared to that of the hay group (*p* = 0.005). In addition, in ileal digesta, the HG diet increased the concentrations of TVFA (*p* = 0.007), propionate (*p* = 0.013), butyrate (*p* = 0.008), valerate (*p* < 0.001), lactate (*p* = 0.008), and LPS (*p* < 0.001), as compared with the hay diet. Compared to those fed the hay diet, HG‐fed goats have higher amylase activity in jejunal (*p* < 0.001) and ileal (*p* < 0.001) digesta.

**Table 1:** Effects of a high‐grain (HG) diet feeding on the jejunal and ileal physiological parameters in goats[Link]

Items	Jejunum			Ileum		
	Hay	HG	*p*‐Value	Hay	HG	*p*‐Value
pH	5.77 ± 0.12	5.76 ± 0.17	0.971	7.85 ± 0.05	7.52 ± 0.08	0.005
TVFA (μmol/g)	6.25 ± 1.16	11.90 ± 1.54	0.015	20.69 ± 2.91	32.56 ± 1.95	0.007
Acetate (μmol/g)	4.02 ± 0.77	6.11 ± 0.85	0.099	17.13 ± 3.53	24.95 ± 2.36	0.103
Propionate (μmol/g)	1.40 ± 0.25	2.99 ± 0.41	0.008	1.98 ± 0.38	3.43 ± 0.29	0.013
Butyrate (μmol/g)	0.26 ± 0.07	0.98 ± 0.18	0.004	0.51 ± 0.12	1.16 ± 0.38	0.008
Isobutyrate (μmol/g)	0.26 ± 0.07	0.66 ± 0.15	0.035	0.63 ± 0.17	0.82 ± 0.09	0.331
Valerate (μmol/g)	0.10 ± 0.02	0.82 ± 0.52	0.257	0.11 ± 0.02	1.81 ± 0.14	<0.001
Isovalerate (μmol/g)	0.21 ± 0.08	0.35 ± 0.10	0.314	0.33 ± 0.09	0.39 ± 0.09	0.684
Lactate (μmol/g)	1.54 ± 0.25	0.50 ± 0.04	0.008	0.30 ± 0.04	1.29 ± 0.21	0.008
Free LPS (EU/g)	25,305 ± 6,149	33,619 ± 9,982	0.550	7,306 ± 808	48,873 ± 3,557	<0.001
Activity of alpha‐amylase (U/mgprot)	4.53 ± 1.06	18.51 ± 2.12	<0.001	2.16 ± 0.78	16.32 ± 1.86	<0.001

Notes

EU: endotoxin unit; LPS: lipopolysaccharide; TVFA: total volatile fatty acid.

Values are means ± *SEM*,* n* = 5. The data were analyzed by an independent *t* test.

### 3.2 Average richness and diversity of the jejunal and ileal mucosal microbiome

In this study, we detected a total of 360,439 reads in jejunal mucosal samples, with a mean of approximately 36,043 reads per sample (Figure S1a). In ileum mucosal samples, we detected 386,975 reads, resulting in a mean of 38,698 reads per sample (Figure S1b). Based on 97% similarity, there was a mean of 498 OTUs in the jejunal mucosa samples (Figure S1a) and a mean of 447 in the ileum mucosal samples (Figure S1b). In the jejunal mucosal samples, the numbers of OTUs (*p* = 0.002), ACE values (*p* = 0.038), and chao1 values (*p* = 0.027) of the HG diet group were higher than in the hay group. The Shannon values (*p* = 0.110) and Simpson's index values (*p* = 0.236) of the HG and hay groups showed no significant difference. In the ileal mucosal samples, we also found no significant between‐group difference in the numbers of OTUs (*p* = 0.411), ACE values (*p* = 0.875), chao1 values (*p* = 0.516), Shannon values (*p* = 0.292), and Simpson's index values (*p* = 0.615) (Table 2).

**Table 2:** Effect of high‐grain (HG) diet on the diversity and average richness of jejunal and ileal mucosal microbiota at the 3% dissimilarity level[Link]

Parameters	Jejunal mucosa			Ileal mucosa		
	Hay	HG	*p*‐Value	Hay	HG	*p*‐Value
OTUs	160 ± 10	218 ± 8	0.002	212 ± 13	201 ± 2	0.411
Ace	203 ± 18	253 ± 10	0.038	244 ± 21	241 ± 3	0.875
Chao1 value	196 ± 15	243 ± 8	0.027	245 ± 20	232 ± 3	0.516
Shannon value	1.29 ± 0.05	1.42 ± 0.05	0.110	1.41 ± 0.09	1.38 ± 0.04	0.292
Simpson	0.43 ± 0.02	0.39 ± 0.02	0.236	0.40 ± 0.03	0.42 ± 0.02	0.615

ACE: abundance‐based coverage estimator; OTUs: operational taxonomic units.

Values are means ± *SEM*,* n* = 5. The data were analyzed by an independent *t* test.

**Figure 1:**
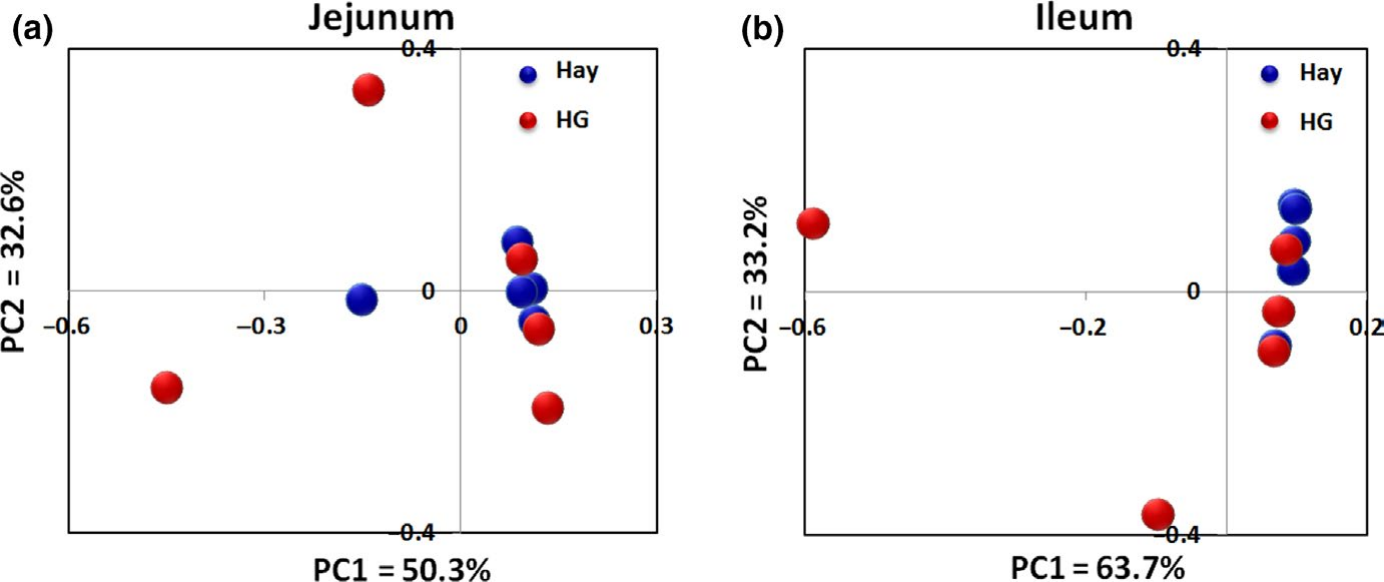
Bray–Curtis distance matrix principal coordinate analysis (PCoA) of mucosa‐attached bacterial microbiota in the jejunum (a) and ileum (b) based on the operational taxonomic unit data

The PCoA plots based on the Bray–Curtis distance matrix showed no significant separation between the samples obtained from the goats fed either diet in jejunum (Figure 1a) and ileum (Figure 1b). The results of the AMOVA revealed no significant between‐group differences in the composition of jejunal and ileal mucosa‐associated microbiota (AMOVA: *F*s = 0.310, *p* = 0.716 in the jejunum and *F*s = 0.798, *p* = 0.859 in the ileum).

### 3.3 Composition of the jejunal and ileal mucosal microbiota

At the phylum level, the results revealed 19 phyla in jejunal mucosal samples and 20 phyla in ileal mucosal samples. In jejunal mucosal samples, most of the sequences belonged to the phyla Proteobacteria (59.33%) and Firmicutes (38.83%), followed by the phyla Actinobacteria (1.19%), Tenericutes (0.30%), and Bacteroidetes (0.24%) (Table 3). The five phyla accounted for more than 99% of the sequences. Rare phyla (<0.01%) were Fusobacteria, Cyanobacteria, Saccharibacteria, unclassified Bacteria, Spirochaetae, Acidobacteria, Fibrobacteres, Chlorobi, Chloroflexi, Deinococcus–Thermus, Latescibacteria, Nitrospirae, Synergistetes, and Verrucomicrobia (Table 3). In ileal mucosal samples, most sequences belonged to the phyla Proteobacteria (56.71%) and Firmicutes (40.30%), followed by the phyla Actinobacteria (2.51%) and Bacteroidetes (0.33%) (Table 4). Bacteria belonging to these four phyla accounted for more than 99% of the sequences (Table 4).

**Table 3:** Effects of high‐grain (HG) feeding on average relative abundance of jejunal mucosa‐attached bacterial microbiota (% of total sequence) at the phylum level in goats (*n* = 5)

Phylum	Relative abundance (%)		*SEM* [Link]	FDR value
	Hay	HG		
Proteobacteria	61.055	57.599	2.299	0.465
Firmicutes	37.355	40.306	2.181	0.465
Actinobacteria	1.092	1.280	0.081	0.347
Tenericutes	0.255	0.354	0.035	0.251
Bacteroidetes	0.164	0.321	0.033	0.009
Fusobacteria	0.022	0.022	0.003	0.917
Cyanobacteria	0.014	0.024	0.004	0.347
Saccharibacteria	0.017	0.017	0.002	0.917
Unclassified Bacteria	0.012	0.054	0.009	0.009
Spirochaetae	0.004	0.001	0.001	0.220
Acidobacteria	0.004	0.007	0.001	0.117
Fibrobacteres	0.003	0.004	0.001	0.169
Chlorobi	0.001	0.001	<0.001	0.881
Chloroflexi	0	0.001	<0.001	0.317
Deinococcus–Thermus	0.001	0.004	0.001	0.131
Latescibacteria	0	0.001	<0.001	0.317
Nitrospirae	0.001	0.002	0.001	0.126
Synergistetes	<0.001	0.003	0.001	0.034
Verrucomicrobia	0	0.001	<0.001	0.317

FDR: false discovery rate.

*SEM*, standard error of the difference of the means. The data were determined using the nonparametric Kruskal–Wallis test.

**Table 4:** Effects of high‐grain (HG) feeding on average relative abundance of ileal mucosa‐attached bacterial microbiota (% of total sequence) at the phylum level in goats (*n* = 5)

Phylum	Relative abundance (%)		*SEM* [Link]	FDR value
	Hay	HG		
Proteobacteria	53.976	59.448	2.449	0.251
Firmicutes	42.827	37.769	2.383	0.251
Actinobacteria	2.797	2.228	0.185	0.175
Bacteroidetes	0.236	0.418	0.091	0.917
Cyanobacteria	0.034	0.027	0.003	0.117
Tenericutes	0.022	0.026	0.004	0.754
Unclassified Bacteria	0.021	0.018	0.003	0.754
Saccharibacteria	0.026	0.023	0.004	0.917
Acidobacteria	0.006	0.003	0.002	0.916
Deinococcus–Thermus	0.001	<0.001	0.001	0.881
Fibrobacteres	0.009	0.003	0.002	0.094
Fusobacteria	0.022	0.022	0.006	0.917
Chlorobi	0.009	0.002	0.003	0.590
Armatimonadetes	0.001	0	<0.001	0.317
Chloroflexi	0.001	0.002	0.001	0.638
Gemmatimonadetes	0.001	0.002	0.001	0.410
Nitrospirae	0.001	0.003	0.001	0.290
Spirochaetae	0.007	0.001	0.002	0.126
Synergistetes	0.002	0	0.001	0.317
Verrucomicrobia	0	0.007	0.003	0.136

FDR: false discovery rate.

*SEM*, standard error of the difference of the means. The data were determined using the nonparametric Kruskal–Wallis test.

At the genus level, the examination of jejunal and ileal mucosal samples revealed 245 and 253 taxa, respectively. *Halomonas* (57.67%), *Lactococcus* (33.98%), *Bacillus* (2.11%), *Streptococcus* (0.97%), *Arthrobacter* (0.89%), *Pseudomonas* (0.52%), and *Carnobacterium* (0.51%) were the dominant genera in jejunal mucosa (Table 5). *Halomonas* (52.50%), *Lactococcus* (34.97%), *Arthrobacter* (2.12%), *Streptococcus* (1.34%), *Bacillus* (0.90%), and *Carnobacterium* (0.81%) were dominant in ileal mucosa (Table 6).

**Table 5:** Effects of high‐grain (HG) feeding on average relative abundance of genus level (% of total sequences) in the jejunal mucosa (*n* = 5)[Link]

Phylum	Genus	Relative abundance (%)		*SEM*	FDR value
		Hay	HG		
Proteobacteria	*Halomonas*	59.267	56.074	2.322	0.465
	*Pseudomonas*	0.697	0.350	0.133	0.251
	*Pelagibacterium*	0.297	0.003	0.063	0.052
	*Acinetobacter*	0.222	0.284	0.018	0.117
	*Aliihoeflea*	0.093	0.133	0.012	0.076
	*Aquabacterium*	0.089	0.134	0.017	0.117
	*Stenotrophomonas*	0.082	0.101	0.005	0.028
	*Comamonas*	0.076	0.084	0.005	0.602
	*Moraxella*	0.037	0.065	0.006	0.009
	*Psychrobacter*	0.028	0.032	0.004	0.917
	*Caulobacter*	0.024	0.027	0.004	0.754
Firmicutes	*Lactococcus*	32.776	35.187	1.929	0.754
	*Bacillus*	1.944	2.274	0.213	0.602
	*Streptococcus*	0.925	1.011	0.050	0.602
	*Carnobacterium*	0.499	0.525	0.036	0.917
	*Solibacillus*	0.367	0.354	0.021	0.602
	*Lysinibacillus*	0.300	0	0.118	0.054
	*Lactobacillus*	0.059	0.132	0.013	0.009
	*Brochothrix*	0.056	0.091	0.011	0.251
	*Leuconostoc*	0.038	0.048	0.004	0.251
	*Enterococcus*	0.033	0.024	0.003	0.465
	*Melissococcus*	0.032	0.031	0.004	0.175
	*Subdoligranulum*	0.031	0.050	0.008	0.347
	*Ruminiclostridium*	0.027	0.032	0.004	0.917
Actinobacteria	*Arthrobacter*	0.804	0.965	0.071	0.465
	*Nesterenkonia*	0.258	0.247	0.016	0.251
Tenericutes	*Mycoplasma*	0.127	0.197	0.023	0.076
	*Ureaplasma*	0.122	0.149	0.013	0.602
Bacteroidetes	*Prevotella*	0.050	0.101	0.010	0.009
	*Empedobacter*	0.022	0.031	0.004	0.347

FDR: false discovery rate; *SEM*, standard error of the difference of the means.

Only top 30 were presented. The data were determined using the nonparametric Kruskal–Wallis test.

**Table 6:** Effects of high‐grain (HG) feeding on average relative abundance of genus level (% of total sequences) in the ileal mucosa (*n* = 5)[Link]

Phylum	Genus	Relative abundance (%)		*SEM*	FDR value
		Hay	HG		
Proteobacteria	*Halomonas*	52.705	58.285	2.450	0.251
	*Pseudomonas*	0.430	0.329	0.041	0.117
	*Comamonas*	0.154	0.149	0.014	0.754
	*Acinetobacter*	0.153	0.056	0.033	0.251
	*Pelagibacterium*	0.133	0.137	0.007	0.347
	*Aquabacterium*	0.096	0.156	0.033	0.917
	*Aliihoeflea*	0.061	0.071	0.005	0.465
Firmicutes	*Lactococcus*	37.657	32.291	2.128	0.175
	Streptococcus	1.366	1.318	0.080	0.602
	*Bacillus*	0.946	0.844	0.064	0.347
	*Carnobacterium*	0.827	0.786	0.050	0.602
	*Solibacillus*	0.488	0.451	0.036	0.754
	*Lysinibacillus*	0.264	0.237	0.016	0.754
	*Lactobacillus*	0.162	0.164	0.009	0.754
	*Ruminococcaceae UCG−014*	0.114	0.049	0.016	0.076
	*Melissococcus*	0.102	0.054	0.015	0.465
	*Subdoligranulum*	0.097	0.046	0.018	0.251
	*Ruminiclostridium*	0.070	0.042	0.010	0.251
	*Christensenellaceae R‐7* group	0.058	0.022	0.010	0.016
	*Enterococcus*	0.048	0.040	0.003	0.251
	*Faecalibacterium*	0.044	0.025	0.007	0.175
	*Leuconostoc*	0.044	0.035	0.005	0.347
	*Lachnoclostridium*	0.036	0.035	0.005	0.754
	*Brochothrix*	0.034	0.031	0.004	0.602
	[*Eubacterium*] *coprostanoligenes* group	0.032	0.025	0.005	0.602
Actinobacteria	*Arthrobacter*	2.390	1.853	0.175	0.117
	*Nesterenkonia*	0.304	0.290	0.033	0.754
Bacteroidetes	*Bacteroides*	0.054	0.127	0.039	0.917
	*Prevotella*	0.045	0.030	0.007	0.347
	*Barnesiella*	0.032	0.038	0.007	0.602

FDR: false discovery rate; *SEM*, standard error of the difference of the means.

Only top 30 were presented. The data were determined using the nonparametric Kruskal–Wallis test.

At the OTU level, the results revealed 498 OTUs in jejunal mucosa and 447 OTUs in ileal mucosa. The relative abundance of the top 30 OTUs of jejunal and ileal mucosa is shown in Tables S3 and S4. These 30 OTUs accounted for 98.14% and 97.64% of the total sequences in jejunal and ileal mucosal samples, respectively. In jejunal mucosa, the results revealed 242 shared OTUs in the two groups and 90 and 166 specific OTUs in the hay and HG groups, respectively. In ileal mucosa, there were 225 shared OTUs and 136 and 86 specific OTUs in the hay group and HG group, respectively.

### 3.4 Changes in the abundance of jejunal and ileal mucosal bacterial phyla, genera, and OTUS

At the phylum level, in jejunal mucosa, the abundance of the phyla Bacteroidetes (FDR = 0.009), Synergistetes (FDR = 0.034), and unclassified Bacteria (FDR = 0.009) was greater in the HG group compared with the hay group (Table 3). However, the results revealed no significant difference between‐group in the percentage of any bacterial phyla in ileal mucosa (FDR > 0.05) (Table 4).

At the genus level, in jejunal mucosa, the HG diet increased the abundance of *Stemotrophomonas* (FDR = 0.028), *Moraxella* (FDR = 0.009), *Lactobacillus* (FDR = 0.009), and *Prevotella* (FDR = 0.009), as compared with the hay diet (Table 5). In ileal mucosa, the HG diet reduced the relative abundance of the *Christensenellaceae R7* group (FDR = 0.010), as compared with the hay diet (Table 6).

At the OTU level, of the top 30 OTUs detected in jejunal mucosa (Table S3), the relative abundance of five OTUs was higher in the HG group (FDR < 0.05), and the relative abundance of one OTU was reduced in ileum mucosa (FDR < 0.05) (Table S4).

### 3.5 Relative mRNA expression of tight junction proteins in the jejunum and ileum

**Figure 2:**
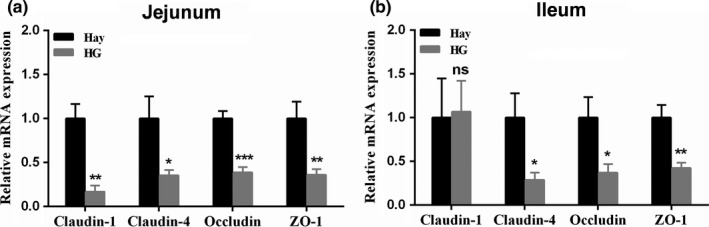
The relative expression of tight junction protein genes in the jejunum (a) and ileum (b) of goats fed hay diet and high‐grain (HG) diet (means ± *SD*,* n* = 5). *0.01 < *p* < 0.05, **0.001 < *p* < 0.01, ***p < 0.001. NS, not significant. The 18S rRNA (a housekeeping gene) mRNA level was used to normalize the relative amount of each studied mRNA, and the $2^{-\Delta \Delta CT}$ method was used to analyze the data. The data were analyzed by an independent *t* test.

Compared with the hay diet, the HG diet significantly downregulated the mRNA expression of claudin‐1 (*p* = 0.001), claudin‐4 (*p* = 0.033), occludin (*p* < 0.001), and ZO‐1 (*p* = 0.010) in jejunal mucosa (Figure 2a). In ileal mucosa, compared with the hay diet, the HG diet decreased the mRNA expression of claudin‐4 (*p* = 0.034), occludin (*p* = 0.033), and ZO‐1 (*p* = 0.004). In contrast, there was no significant between‐group difference in the mRNA expression of claudin‐1 (*p* = 0.905) (Figure 2b).

## 4 DISCUSSION

Over the past few decades, research on the effect of a HG diet on VFA profiles in the rumen and hindgut of ruminants has been widely explored (Li et al., 2012; Metzler‐Zebeli et al., 2013). However, little information is available on changes of VFA pattern in the small intestine during HG feeding. In the present study, we found that the HG diet increased the TVFA, propionate, and butyrate concentration in digesta of the jejunum and ileum. These results were also consistent with the findings of our previous study (Mao et al., 2013), which showed that, compared with 0% corn diet feeding, 50% corn diet feeding increased the levels of acetate, butyrate, and total VFA in the ileum digesta of goats. Our previous study has showed that HG feeding increased the concentrations of acetate, propionate, butyrate, and total VFAs in the rumen fluid of goats (Zhang, Ye, Liu, & Mao, 2017). Therefore, the higher VFA in small intestine of HG‐fed goats may be due to the facts that the excessive VFA escaped from the rumen into the small intestine of goats. In the current study, due to the insufficient small intestinal digesta and large amount of water in digesta, we are not able to measure the amount of starch in small intestinal digesta. Therefore, we could not demonstrate directly that there were more rumen bypass and starch inflow into the small intestine of HG‐fed goats. However, we found that the HG diet significantly increased the activity of alpha‐amylase in the jejunum and ileum. In the small intestine, amylase breaks down starch into glucose, which is absorbed by the animal body (Nozière, Steinberg, Silberberg, & Morgavi, 2014). Swanson et al. (2000) confirmed that high starch and high energy diets can increase the activity of alpha‐amylase and alpha‐amylase protein in lambs. Thus, we inferred that there may be more rumen bypass and starch inflow into the small intestine in HG‐fed goats, which resulted in higher VFA concentrations.

Mucosa‐associated bacteria in the gastrointestine play a vital role in regulating the barrier function and immune system (Kelly et al., 2016). Previous researchers used the high‐throughput sequencing method to study the diversity and composition of epithelium‐associated microbial communities in the rumen and hindgut (colon and cecum) of goats (Jiao, Huang, Zhou, & Tan, 2015; Liu et al., 2014; Ye et al., 2016). However, only a few studies have utilized molecular tools to examine microbial composition and diversity in the mucosa‐associated microbiota of the small intestine in ruminants (Malmuthuge et al., 2012; Malmuthuge, Griebel, & Guan, 2014). Our data provide a detailed picture of the jejunal and ileal mucosa‐associated microbiota in goats. At the phylum level, the phyla Proteobacteria (59.33% in jejunal mucosa and 56.71% in ileal mucosa) and Firmicutes (38.83% in jejunal mucosa and 40.30% in ileal mucosa) dominated the bacterial community of jejunal and ileal mucosa. These two phyla are also highly abundant in the ileum of juvenile goats (Jiao et al., 2016) and jejunum and ileum of Sika Deer (Li et al., 2018). The common distribution of these microbiota from different ruminants indicates their general importance in specific function of small intestine in ruminants. The present data are somehow inconsistent with the results of Malmuthuge et al. (2014), who reported that the jejunal and ileal mucosa of calves mainly consisted of Bacteroidetes (23.6% in jejunal mucosa and 33.7% in ileal mucosa), Firmicutes (36.2% in jejunal mucosa and 31.8% in ileal mucosa), and Proteobacteria (29.2% in jejunal mucosa and 24.4% in ileal mucosa) (Malmuthuge et al., 2014). The discrepancy may be explained by the difference in the nutrient composition of the diets, host species, and animal ages. At the genus level, *Halomonas* (57.67%), *Lactococcus* (33.98%), and *Bacillus* (2.11%) were the dominant genera in jejunal mucosa, and *Halomonas* (52.50%), *Lactococcus* (34.97%), *Arthrobacter* (2.12%), and *Streptococcus* (1.34%) were dominant in ileal mucosa. These results showed that *Halomonas* was the most dominant genus associated with jejunal and ileal mucosa in goats. *Halomonas* has been demonstrated to inhibit enteric LPS‐induced human monocyte activation producing several pro‐inflammatory cytokines (Ialenti et al., 2006). It is speculated that *Halomonas* may play a role in facilitating the gut immune development. However, this hypothesis needs to be documented in future study through in vitro cultivation approach. The common distribution of this genus in jejunum and ileum indicates its specific importance in immune function of small intestine. Our results showed that Bacillus is dominant in jejunal mucosa but not in ileal mucosa. Previous studies have demonstrated that many *Bacillus* species are able to secrete large quantities of amylase (Asgher, Asad, Rahman, & Legge, 2007) and protease (Puri, Beg, & Gupta, 2002) to facilitate starch and protein degradation. Therefore, the higher relative abundance of *Bacillus* in the jejunum may be linked with its specific function in digestion of starch and protein.

Previous studies showed that a long‐term HG diet appeared to disrupt the balance of the ruminal microbiota and affect the rumen health of dairy cattle and goats (Hua et al., 2017; Wetzels, et al., 2017). In the present study, the HG diet increased the richness of bacterial communities of jejunal mucosa, but it seemed to have no apparent effect on the richness of bacterial communities of ileal mucosa, with no significant between‐group difference observed. These results indicate that the HG diet had a regional effect on the richness of the bacterial microbiota of the small intestine. These different effects of gut site may be due to the functional heterogeneity of jejunum and ileum in goats. Furthermore, the different changes in VFA patterns in the jejunum and ileum may also somehow affect the richness of the bacteria.

In addition, the current study revealed that the HG diet increased the abundance of *Prevotella*,* Lactobacillus*,* Stenotrophomonas*, and *Moraxella* in jejunal mucosa. Of these genera, *Prevotella* was the most abundant genus in the phylum Bacteroidetes of jejunal mucosal microbiota. A previous study reported that members of *Prevotella* utilized various substrates and that they played an essential role in plant starch and protein digestion (Avgustin, Wallace, & Flint, 1997). Research also showed that this bacterial genus exhibited a remarkable degree of phenotypic diversity, enabling it to occupy various ecological niches within the rumen (Avgustin et al., 1997). Our results shed further light on the presence of *Prevotella* in the jejunal mucosa, especially in animals fed a HG diet. Additionally, Respondek, Goachet, and Julliand (2008) showed that the population of *Lactobacillus*, as the main lactate producer, was increased in the hindgut after a large intake of starch in horses. Thus, the higher proportion of *Lactobacillus* in jejunal mucosal microbiota may be associated with increased starch content in HG group. Jenkins, Waite, Mansfield, Kim, and Pluske (2015) reported that species belonging to the Christensenellaceae family were common in the rumen and that these species played an essential role in maintaining gastrointestinal structure and function. We also detected a higher proportion of the *Christensenellaceae R7* group (Firmicutes, butyrate producer) in ileal mucosal microbiota of goats fed a HG diet. On the one hand, this result somehow explains the higher butyrate concentration in HG group; on the other hand, the enrichment of these genera may be an adaptation response to HG feeding.

Many previous studies have reported that a HG diet increased the number of pathogens in the rumen and feces (Berry et al., 2006; Khafipour, Li, Plaizier, & Krause, 2009; Russell & Rychlik, 2001). When enteric pathogens pass the mucosal barrier, intestinal inflammation occurs (Pedron & Sansonetti, 2008). Our findings revealed that HG feeding increased the percentage of *Stenotrophomonas* and *Moraxella* in the jejunal mucosa. Of these two genera, *Stenotrophomonas* spp. (a Gram‐negative bacterium) has been linked to severe mucositis, neutropenia, diarrhea, and life‐threatening chronic enteritis (Apisarnthanarak et al., 2003; Rausch et al., 2011). Moraxella spp. are common mucosal pathogens in patients with chronic obstructive pulmonary disease (Murphy, Brauer, Aebi, & Sethi, 2005). The relatively higher abundance of these two genera in the HG group indicates that HG feeding may have a negative effect on the jejunal health of goats. It is very interesting that we found that it has higher percentage of *Stenotrophomonas* and *Moraxella* in jejunum, but these two genera were not seen in ileum samples. Many previous studies have demonstrated that *Stenotrophomonas* (Lukas, Simunek, Mrazek, & Kopecny, 2010) and *Moraxella* (Camboim et al., 2012) were also identified in the rumen of cattle and goats. Therefore, we inferred that these pathogens in jejunum may origin from the rumen and when they entering into the ileum, the changed chemical environment may be not fit for their survival in the ileum.

The mucosal barrier of the small intestine of the host provides defense against the entry of harmful pathogens and toxins from the luminal digesta. Changes in the composition of jejunal and ileal mucosal microbiota and the chemical environment may affect the integrity of the mucosal barrier of the small intestine (Ulluwishewa et al., 2011). Compared with ruminal epithelium, which has multicellular layers, the intestinal epithelium of ruminants consists only of a single layer of epithelial cells (Plaizier et al., 2012), making it more susceptible to be damaged by an abnormal chemical environment or bacteria. In the present study, the HG diet reduced the relative expression of claudin‐4, occludin, and ZO‐1 in the ileum, as shown by the real‐time PCR results. The HG diet also lowered the expression of claudin‐1, claudin‐4, occludin, and ZO‐1 genes in jejunal mucosa. These results suggested that the HG diet may have damaged the mucosal barrier of the small intestine in goats. Our data are somewhat consistent with those of an earlier study (Liu et al., 2013), which found that HG feeding damaged the ruminal epithelium and downregulated the expression of epithelial tight junction proteins in goats. Small intestine is mainly responsible for protein digestion and absorption in ruminants. Previous study has shown that mucosa‐associated bacteria play vital roles in nutrients transport (Kleessen & Blaut, 2005). Therefore, the changes in bacterial composition may disturb the amino acid absorption, which may somehow affect the synthesis of proteins related to tight junctions and immune function and finally resulted in dysfunction of barrier and immune in the small intestine. Presumably, a lower pH and higher level of LPS in digesta may also contribute to the damage of the mucosal barrier of the ileum. Moreover, previous studies showed that the pathogenic bacteria *Stenotrophomonas* and *Moraxella* might induce the release of inflammatory cytokines and result in mucosal damage in colitis (Elinav et al., 2011; Saleh & Trinchieri, 2011). Overall, the findings of the present study indicate that, in the small intestine of animals fed a HG diet, impaired epithelial barrier function may be related to changes in the composition of mucosal‐attached microbiota. Furthermore, a combination of alterations in the chemical environment and increases in LPS levels in response to a HG diet may have adverse effects on small intestinal health.

In summary, our research revealed increased concentrations of TVFA and butyrate in jejunal and ileal digesta, as well as decreased ileal pH levels, in goats fed a HG diet, as compared with goats fed a hay diet. Our findings also showed that feeding goats a HG diet increased the proportion of some potential pathogens (*Stenotrophomonas* and *Moraxella*) in jejunal mucosa. In ileal mucosa, the HG diet increased the relative abundance of the Christensenellaceae R7 group. The HG diet also decreased mRNA expression of tight junction proteins in the jejunum and ileum. Overall, our findings indicate that feeding goats a HG diet changes the VFA patterns, alters the relative abundance of some specific bacteria associated with mucosa, and results in downregulation of genes related to tight junction proteins in the small intestine of goats.

## CONFLICT OF INTEREST

The authors declare that there is no conflict of interest.

## AUTHORS CONTRIBUTION

J. L., C. X., and D. S. carried out all the experiments. J. L., C. X., and S. M. analyzed the data. S. M., J. L., and W. Z. were responsible for study design. J. L. and S. M. were responsible for writing the manuscript.

## Data Availability

The sequencing data for all the samples analyzed in this study were deposited in the Sequence Read Archive (SRA) under accession SRP157077.
