# Supplementary material for: Impact of high‐grain diet feeding on mucosa‐associated bacterial community and gene expression of tight junction proteins in the small intestine of goats
Source: Microbiologyopen. 2018 Oct 24;8(6):e00745. doi: 10.1002/mbo3.745 (PMC6562116; doi:10.1002/mbo3.745)
Supplement: Supplementary file 1 [file MBO3-8-e00745-s001.docx]

**Table S1** Ingredients and nutrient composition of the experimental diets.

| Items | Hay High Grain | |
| --- | --- | --- |
| Ingredients (% of DM) |  | |
| *Leymus chinensis* | 80.00 | 18.00 |
| Medicago sativa hay | 16.00 | 7.00 |
| Corn | 0.00 | 20.00 |
| Wheat | 0.00 | 36.50 |
| Soybean meal | 0.00 | 15.00 |
| Limestone meal | 0.70 | 1.00 |
| Calcium phosphate dibasic | 1.80 | 1.00 |
| Salt | 0.50 | 0.50 |
| Premix^1^ | 1.00 | 1.00 |
| Total | 100.00 | 100.00 |
| Nutrient levels |  | |
| Metabolic energy (MJ/kg) | 8.32 | 11.56 |
| Crude fiber (%) | 30.13 | 10.13 |
| Crude protein (%) | 10.16 | 17.19 |
| Neutral detergent fiber (%) | 56.84 | 22.75 |
| Acid detergent fiber (%) | 35.70 | 12.78 |

^1^Contained 16% calcium carbonate, 102 g/kg of Zn, 47 g/kg of Mn, 26 g/kg of Cu, 1,140 mg/kg of I, 500 mg/kg of Se, 340 mg/kg of Co, 17,167,380 IU/kg of vitamin A, 858,370 IU/kg of vitamin D, and 23,605 IU/kg of vitamin E.

| Gene Name | Forward and Reverse Sequences | Reference | Amplicon Size, bp | Effeciency, % |
| --- | --- | --- | --- | --- |
| *Claudin-1* | F: CACCCTTGGCATGAAGTGTA | Liu et al. (2013) | 216 | 102 |
|  | R: AGCCAATGAAGAGAGCCTGA |  |  |  |
| *Claudin-4* | F: AAGGTGTACGACTCGCTGCT | Liu et al. (2013) | 238 | 103 |
|  | R: GACGTTGTTAGCCGTCCAG |  |  |  |
| *Occludin* | F: GTTCGACCAATGCTCTCTCAG | Liu et al. (2013) | 200 | 93 |
|  | R: CAGCTCCCATTAAGGTTCCA |  |  |  |
| *ZO-1* | F: CGACCAGATCCTCAGGGTAA | Liu et al. (2013) | 163 | 95 |
|  | R: AATCACCCACATCGGATTCT |  |  |  |
| *18S rRNA* | F: TTCCCAGTAAGTGCGGGTCATAAG | - | 152 | 98 |
|  | R: AATCGGTAGTAGCGACGGGCGGTG |  |  |  |

**Table S2** Primers used for quantitative real-time PCR analysis.

**Table S3** Effects of high grain (HG) feeding on average relative abundance of operational taxonomic units (OTUs) level (% of total sequences) in the jejunal mucosa (n=5)^1^

| Phylum | OTUs | Genus | Relative abundance (%) | | SEM^2^ | *FDR^3^* value |
| --- | --- | --- | --- | --- | --- | --- |
|  |  |  | Hay | HG |  |  |
| Proteobacteria | OTU511 | *Acinetobacter* | 0.180 | 0.198 | 0.013 | 0.754 |
|  | OTU624 | *Acinetobacter* | 0.014 | 0.046 | 0.007 | 0.009 |
|  | OTU779 | *Aliihoeflea* | 0.090 | 0.133 | 0.012 | 0.076 |
|  | OTU158 | *Aquabacterium* | 0.085 | 0.134 | 0.017 | 0.117 |
|  | OTU186 | *Comamonas* | 0.067 | 0.074 | 0.005 | 0.251 |
|  | OTU182 | *Halomonas* | 59.720 | 56.074 | 2.322 | 0.465 |
|  | OTU482 | *Moraxella* | 0.040 | 0.065 | 0.006 | 0.009 |
|  | OTU107 | *Pseudomonas* | 0.185 | 0.299 | 0.040 | 0.076 |
|  | OTU609 | *Stenotrophomonas* | 0.078 | 0.088 | 0.005 | 0.251 |
| Firmicutes | OTU20 | *Bacillus* | 0.002 | 0.032 | 0.010 | 0.245 |
|  | OTU404 | *Bacillus* | 1.541 | 1.641 | 0.181 | 0.465 |
|  | OTU757 | *Bacillus* | 0.524 | 0.551 | 0.048 | 0.754 |
|  | OTU255 | *Brochothrix* | 0.054 | 0.091 | 0.011 | 0.251 |
|  | OTU645 | *Carnobacterium* | 0.465 | 0.525 | 0.036 | 0.917 |
|  | OTU116 | *Lactobacillus* | 0.027 | 0.039 | 0.005 | 0.117 |
|  | OTU374 | *Lactobacillus* | 0.008 | 0.035 | 0.006 | 0.009 |
|  | OTU401 | *Lactococcus* | 5.952 | 6.486 | 0.378 | 0.602 |
|  | OTU630 | *Lactococcus* | 26.461 | 28.701 | 1.552 | 0.754 |
|  | OTU533 | *Melissococcus* | 0.032 | 0.031 | 0.004 | 0.175 |
|  | OTU522 | *Solibacillus* | 0.366 | 0.354 | 0.021 | 0.602 |
|  | OTU103 | *Streptococcus* | 0.430 | 0.526 | 0.031 | 0.117 |
|  | OTU434 | *Streptococcus* | 0.430 | 0.410 | 0.024 | 0.602 |
|  | OTU448 | *Streptococcus* | 0.037 | 0.042 | 0.002 | 0.076 |
| Actinobacteria | OTU220 | *Arthrobacter* | 0.050 | 0.212 | 0.049 | 0.028 |
|  | OTU292 | *Arthrobacter* | 0.750 | 0.753 | 0.048 | 0.917 |
|  | OTU813 | *Nesterenkonia* | 0.248 | 0.242 | 0.015 | 0.251 |
| Tenericutes | OTU714 | *Mycoplasma* | 0.124 | 0.166 | 0.019 | 0.117 |
|  | OTU17 | *Ureaplasma* | 0.123 | 0.149 | 0.013 | 0.602 |
| Bacteroidetes | OTU192 | *Empedobacter* | 0.026 | 0.031 | 0.004 | 0.347 |
| Unclassified Bacteria | OTU604 | Unclassified Bacteria | 0.002 | 0.032 | 0.006 | 0.008 |

^1^only top 30 were presented. The data were determined using the nonparametric Kruskal–Wallis test.

^2^SEM, standard error of the difference of the means.

^3^FDR, false discovery rate.

**Table S4** Effects of high grain (HG) feeding on average relative abundance of operational taxonomic units (OTUs) level (% of total sequences) in the ileal mucosa (n=5)^1^

| Phylum | OTUs | Classification | Relative abundance (%) | | SEM^2^ | *FDR^3^* value |
| --- | --- | --- | --- | --- | --- | --- |
|  |  |  | Hay | HG |  |  |
| Proteobacteria | OTU511 | *Acinetobacter* | 0.113 | 0.026 | 0.029 | 0.251 |
|  | OTU779 | *Aliihoeflea* | 0.061 | 0.071 | 0.005 | 0.465 |
|  | OTU158 | *Aquabacterium* | 0.096 | 0.156 | 0.033 | 0.917 |
|  | OTU183 | *Bradyrhizobium* | 0.009 | 0.002 | 0.002 | 0.131 |
|  | OTU186 | *Comamonas* | 0.153 | 0.146 | 0.014 | 0.602 |
|  | OTU182 | *Halomonas* | 52.705 | 58.285 | 2.451 | 0.251 |
|  | OTU160 | *Pelagibacterium* | 0.133 | 0.137 | 0.007 | 0.347 |
|  | OTU107 | *Pseudomonas* | 0.141 | 0.112 | 0.012 | 0.076 |
|  | OTU109 | *Pseudomonas* | 0.068 | 0.007 | 0.031 | 0.754 |
|  | OTU369 | *Pseudomonas* | 0.221 | 0.209 | 0.010 | 0.602 |
| Firmicutes | OTU404 | *Bacillus* | 0.404 | 0.354 | 0.027 | 0.175 |
|  | OTU757 | *Bacillus* | 0.473 | 0.422 | 0.035 | 0.465 |
|  | OTU215 | *Candidatus Arthromitus* | 0.008 | 0.002 | 0.001 | 0.047 |
|  | OTU645 | *Carnobacterium* | 0.827 | 0.786 | 0.050 | 0.602 |
|  | OTU184 | *Christensenellaceae R-7 group* | 0.010 | 0 | 0.004 | 0.136 |
|  | OTU116 | *Lactobacillus* | 0.085 | 0.083 | 0.004 | 0.917 |
|  | OTU401 | *Lactococcus* | 6.941 | 6.067 | 0.401 | 0.347 |
|  | OTU630 | *Lactococcus* | 30.715 | 26.224 | 1.730 | 0.175 |
|  | OTU627 | *Lysinibacillus* | 0.264 | 0.237 | 0.016 | 0.754 |
|  | OTU533 | *Melissococcus* | 0.102 | 0.054 | 0.015 | 0.465 |
|  | OTU118 | *Romboutsia* | 0.019 | 0.121 | 0.024 | 0.117 |
|  | OTU522 | *Solibacillus* | 0.488 | 0.451 | 0.036 | 0.754 |
|  | OTU103 | *Streptococcus* | 0.690 | 0.565 | 0.046 | 0.175 |
|  | OTU434 | *Streptococcus* | 0.546 | 0.479 | 0.040 | 0.465 |
|  | OTU448 | *Streptococcus* | 0.095 | 0.076 | 0.011 | 0.251 |
|  | OTU79 | Unclassified Aerococcaceae | 0.008 | 0.003 | 0.002 | 0.116 |
| Actinobacteria | OTU220 | *Arthrobacter* | 0.159 | 0.107 | 0.016 | 0.117 |
|  | OTU292 | *Arthrobacter* | 2.231 | 1.745 | 0.161 | 0.117 |
|  | OTU813 | *Nesterenkonia* | 0.291 | 0.281 | 0.032 | 0.917 |
| Fibrobacteres | OTU678 | *Fibrobacter* | 0.007 | 0.003 | 0.001 | 0.142 |

^1^only top 30 were presented. The data were determined using the nonparametric Kruskal–Wallis test.

^2^SEM, standard error of the difference of the means.

^3^FDR, false discovery rate.

Liu et al., Figure S1


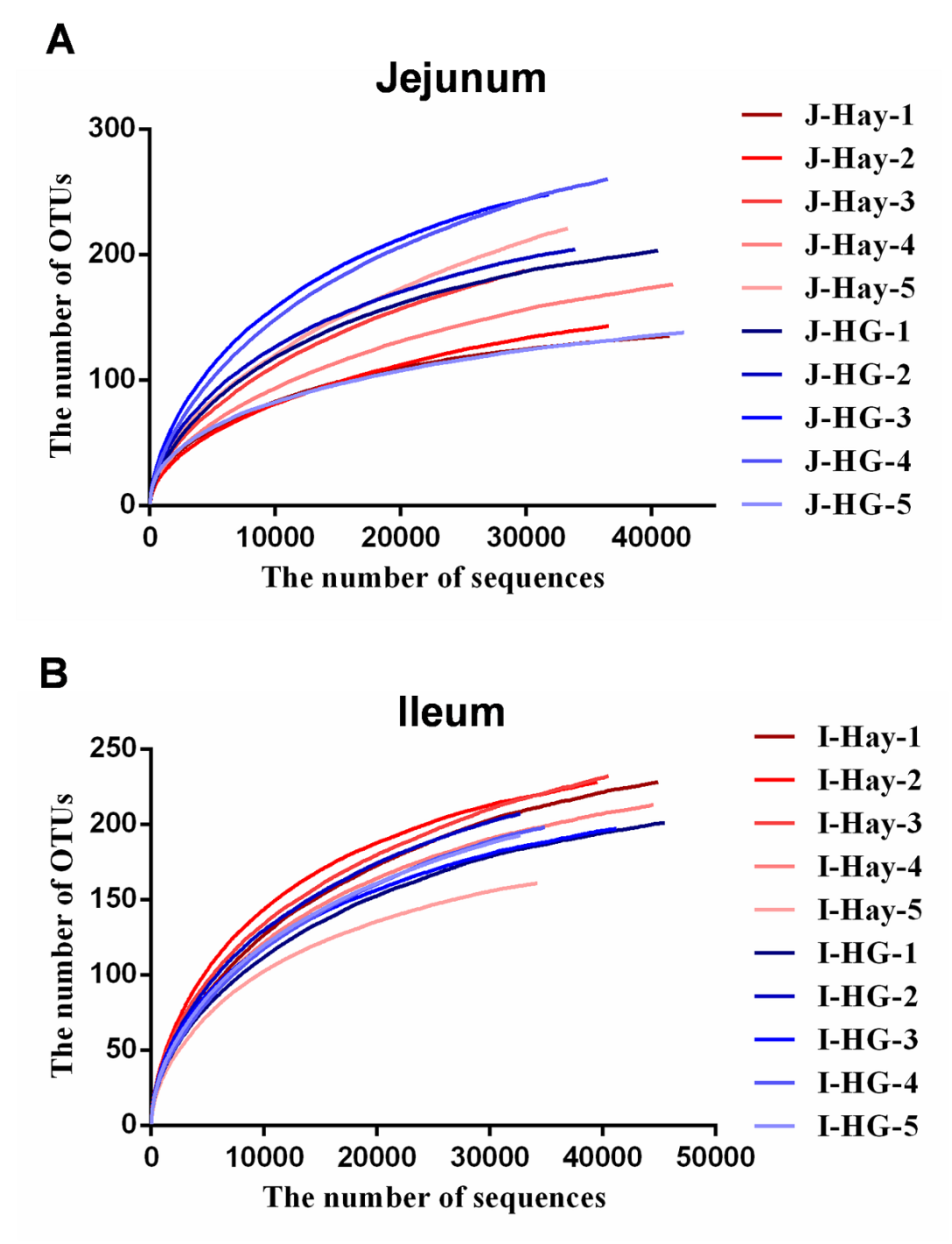


**Figure S1** Rarefaction curves based on operational taxonomic unit (OTUs, 3% divergence) for each jejunal mucosal (A) and ileal mucosal (B) sample. Jejunum mucosa of Hay group: J-Hay-1, J-Hay-2, J-Hay-3, J-Hay-4, J-Hay-5; Jejunum mucosa of High grain (HG) group: J-HG-1, J-HG-2, J-HG-3, J-HG-4, J-HG-5. Ileum mucosa of Hay group: I-Hay-1, I-Hay-2, I-Hay-3, I-Hay-4, I-Hay-5; Ileum mucosa of HG group: I-HG-1, I-HG-2, I-HG-3, I-HG-4, I-HG-5.

Liu et al., Figure S2

**

**

**Figure S2**. Distribution of phyla for each sample.
